# Global relative species loss due to first‐generation biofuel production for the transport sector

**DOI:** 10.1111/gcbb.12597

**Published:** 2019-03-06

**Authors:** Pieter M. F. Elshout, Rosalie van Zelm, Marijn van der Velde, Zoran Steinmann, Mark A. J. Huijbregts

**Affiliations:** ^1^ Department of Environmental Science Institute for Water and Wetland Research, Radboud University Nijmegen Nijmegen The Netherlands; ^2^ European Commission, Joint Research Centre Ispra Italy

**Keywords:** biodiversity, biofuels, global relative species loss, greenhouse gas emissions, land occupation, land transformation, water use

## Abstract

The global demand for biofuels in the transport sector may lead to significant biodiversity impacts via multiple human pressures. Biodiversity assessments of biofuels, however, seldom simultaneously address several impact pathways, which can lead to biased comparisons with fossil fuels. The goal of the present study was to quantify the direct influence of habitat loss, water consumption and greenhouse gas (GHG) emissions on potential global species richness loss due to the current production of first‐generation biodiesel from soybean and rapeseed and bioethanol from sugarcane and corn. We found that the global relative species loss due to biofuel production exceeded that of fossil petrol and diesel production in more than 90% of the locations considered. Habitat loss was the dominating stressor with Chinese corn, Brazilian soybean and Brazilian sugarcane having a particularly large biodiversity impact. Spatial variation within countries was high, with 90th percentiles differing by a factor of 9 to 22 between locations. We conclude that displacing fossil fuels with first‐generation biofuels will likely negatively affect global biodiversity, no matter which feedstock is used or where it is produced. Environmental policy may therefore focus on the introduction of other renewable options in the transport sector.

## 1 INTRODUCTION

Over the last several decades, various national and local incentives have promoted the use of renewable energy sources as a step toward more sustainable energy use. In major renewable energy markets such as the US, Brazil and the EU, bioenergy from biomass is the most important renewable energy source, and further growth is expected in all sectors including the transport sector (IEA, 2018). However, an increasing demand for biomass can only partly be met by intensifying existing agriculture, and will thus require expansion of the global agricultural area (Beringer, Lucht, & Schaphoff, 2011; Helmut et al., 2013). A potential downside of such expansion is the potential loss of species when natural vegetation is transformed into croplands (Dale, Kline, Wiens, & Fargione, 2010; Elshout, Zelm, Karuppiah, Laurenzi, & Huijbregts, 2014; Strona et al., 2018). Additionally, expansion or intensification of agricultural land use may require the extraction of extra surface water to irrigate the feedstocks (Gerbens‐Leenes, Hoekstra, & Meer, 2009). Therefore, biofuel production may negatively affect the freshwater biodiversity as well as the wetland species that depend on surface water (Verones, Pfister, Zelm, & Hellweg, 2017; Vörösmarty et al., 2010).

Furthermore, to provide fertile soils, the removal of natural biomass and the disturbance of the original soil carbon dynamics (e.g. due to tillage) will induce the release of greenhouse gases (GHGs) into the atmosphere (Searchinger et al., 2008). Additional GHGs are emitted during crop cultivation as a result of farm machinery use, cropland fertilization and irrigation, and other processes that require fossil fuels (Lal, 2004; Snyder, Bruulsema, Jensen, & Fixen, 2009). Various studies have provided evidence that switching to first‐generation biofuels may effectively result in an *increase* in GHG emissions (Don, Osborne, & Hastings, 2011; Fargione, Hill, Tilman, Polasky, & Hawthorne, 2008; Hoefnagels, Smeets, & Faaij, 2010; Immerzeel, Verweij, Hilst, & Faaij, 2013; Searchinger et al., 2008), and could thereby contribute to climate change rather than reduce it.

According to Verones, Moran, Stadler, Kanemoto, and Wood (2017), land use, water use and GHG emissions are the three main drivers of ecosystem damage. Hence, when assessing the impact of displacing fossil fuels with biofuels on biodiversity, it is important to consider all three drivers. Previously, the global impact of (agricultural) land transformation on biodiversity has been quantified, typically based on species‐area relationships (De Baan, Mutel, Curran, Hellweg, & Köllner, 2013; Chaudhary, Verones, Baan, & Hellweg, 2015; Schmidt, 2008). To date, only a few studies have applied such models to the case of biofuels. Chaudhary et al. (2015) analysed biodiversity impacts of bioethanol production in different areas of the world showing that sugarcane production in Brazil results in a greater species loss than sugar beet production in France and maize (grain or stover) production in the USA. However, they did not address the additional impacts of water use and GHG emissions on biodiversity. Danielsen, Beukema, and Burgess (2009) compared species richness in natural tropical ecosystems with species richness in oil palm plantations to quantify the impact of oil‐palm‐related land transformation. While they also estimated the CO_2_ emissions related to land transformation, they did not quantify the impact of climate change on biodiversity. Strona et al. (2018) concluded that large‐scale expansion of oil palm cultivation in Africa will have unavoidable negative effects on primates, as there are very few areas that combine a high productivity with low biodiversity importance. Gibon, Hertwich, Arvesen, Singh, and Verones (2017) and Van Zelm et al. (2014) carried out comprehensive assessments of the impacts of GHG emissions and land use (along with acidification and toxicity, but no water use) related to electricity generation and wood‐based biofuel production, respectively. A study that assesses the biodiversity loss related to first‐generation biofuel production worldwide is currently lacking.

The goal of the present study was to quantify the impact on global relative species richness of current first‐generation biofuel production. The selected biofuels included bioethanol from corn and sugarcane, a potential replacement for fossil petrol, and biodiesel from rapeseed and soybean, an alternative to fossil diesel. The focus area included predominant biofuel‐producing countries, namely, the USA (corn and soybean); Brazil (soybean and sugarcane); China (corn); and several European countries including Austria, France, Germany, Italy and Poland (all rapeseed). We assessed the three most important stressors: (a) habitat loss due to land use, (b) habitat loss due to water use and (c) climate change due to GHG emissions. For GHG emissions we not only included the potential species loss in the current situation, but also in future years, as GHG emissions are not directly removed from the atmosphere. We used a default of species loss integrated over a time horizon of 100 years. We analysed two scenarios where biofuels are being produced respectively with and without accounting for the conversion of natural grassland or forest. The scenario “without land conversion” accounts for potential global species loss in the current situation due to cropping activities (e.g. irrigation, fertilizer application) and land occupation compared to the natural state. The scenario “with land conversion” adds biodiversity impacts due to initial loss of carbon after land conversion and the recovery time required for the cropland to go back to the natural state.

## 2 MATERIAL AND METHODS

The biodiversity impact related to biofuel production is expressed as the global potentially disappeared fraction (PDF) of species per MJ of bioenergy produced every year. We quantified this potential global loss of species due to biofuel production by using the LC‐IMPACT method (Verones et al., 2016). LC‐IMPACT distinguishes itself from other life cycle impact assessment methods including the ReCiPe method (Huijbregts et al., 2016), which typically quantified potential species losses at the local scale. The total biodiversity impact was divided in two components, i.e. occupation and transformation. Biodiversity impacts were allocated between the biofuels and by‐products (e.g., corn stover, sugarcane bagasse) based on their respective market values. The allocation factors were collected from Wang, Huo, and Arora (2011) and are shown in Table S1. Throughout our analysis, we assume that natural vegetation (either grassland or forest) would be the counterfactual to the croplands being transformed and occupied for feedstock cultivation.

### 2.1 Occupation

The impact of land occupation from crop *x* cultivated in location *i* under management strategy *j* (*I_occ,x,i,j_* in PDF·yr MJ^−1^) was calculated as the sum of the fraction of species lost due to habitat loss, water stress, and GHG emissions:

$$I_{occ,x,i,j}=\frac{1}{{CEF}_{x,i,j}}∙\left({BF}_{HL,occ,i,j}+\left(W_{occ,x,i,j}∙{BF}_{WS,i}\right)+\left(M_{occ,GHG,x,i,j}∙{BF}_{GHG}\right)\right)$$

where CEF is the crop‐to‐energy conversion efficiency (in MJ m^−2^ yr^−1^); BF_HL,occ_ is the terrestrial biodiversity impact factor for species loss caused by land occupation (in PDF m^‐2^); *W*_occ_ is the amount of water used during feedstock cultivation (in m^3^ m^−2^ yr^−1^); BF_WS_ is the biodiversity impact factor for species loss caused by water stress (in PDF m^−3^); *M*_occ,GHG_ is the GHG emission during biofuel production (in kg CO_2_eq m^−2^ yr^−1^); and BF_GHG_ is the terrestrial biodiversity impact factor per unit of GHG emission (in PDF yr kg CO_2_eq^−1^).

The CEF was calculated as,

$${CEF}_{x,i,j}=Y_{x,i,j}∙{CBF}_{x}∙{EC}_{x}$$

where *Y* is the crop yield (in kg crop m^−2^ yr^−1^); CBF is the crop‐to‐biofuel conversion factor (in kg biofuel kg crop^−1^); and EC is the biofuel energy content (in MJ kg biofuel^−1^).

The BF_GHG_ is calculated as,

$${BF}_{GHG}={IAGTP}_{{CO}_{2}}∙{EF}_{terr}$$

where IAGTP is the time‐integrated absolute global temperature potential of 1 kg of CO_2_ emitted (°C·yr kg CO_2_eq^−1^), and EF_terr_ is the effect factor representing the increase in global PDF due to an increase in global mean temperature (PDF °C^−1^). The IAGTP varies with the time horizon. We used a 100‐year time horizon as default and applied the long‐term effect at a 1,000‐year time horizon as a sensitivity check.

### 2.2 Transformation

The biodiversity impact related to transformation (*I*_trans_ *_,x,i,j_* in PDF yr MJ^−1^) was calculated as the sum of species lost caused by initial GHG emissions directly after natural land conversion and the habitat loss due to destruction of the original ecosystem:

$$I_{trans,x,i,j}=\frac{1}{{CEF}_{x,i,j}∙PT}∙\left(M_{trans,GHG,i}\;∙\;{BF}_{GHG}+{BF}_{HL,trans,i,j}\right)$$

where *M*_trans,GHG_ is the GHG emission resulting from land transformation (in kg CO_2_eq m^−2^); BF_HL,trans_ is the biodiversity impact factor per m^2^ of transformed land (in PDF yr m^−2^); and PT is the plantation time (in years). The default plantation period was set to 30 years, which means that we allocated 3.3% (1/30) of the land conversion impacts to the amount of crops produced in a year. As a sensitivity check, we also calculated transformation impacts for a plantation period of 100 years.

### 2.3 Crop data

Locations of crop cultivation were collected from SPAM ( http://mapspam.info), a model that simulates agriculture at a resolution of 10 km by 10 km at the equator and reduces grid‐cell sizes as the distance to the equator increases. It distinguishes among four farm management strategies, which were reduced to three strategies by combining the farms under low input, rain‐fed management and those under subsistence, rain‐fed management into one low input—no irrigation category. The other two farm management strategies are high input—no irrigation and high input—irrigated. We assume that any agricultural arable land within a country producing the crops of interest can supply the feedstock for that country's biofuel production. Furthermore, we do not include international trade of biofuel feedstocks. Spatially explicit crop yields were collected from SPAM, while crop‐to‐biofuel conversion efficiencies and biofuel energy contents were based on the ecoinvent database (Weidema et al., 2013; Wernet et al., 2016) and its documentation (Jungbluth, 2007).

### 2.4 Carbon stock data

The GHG emissions resulting from land transformation (*M*_trans,GHG_) were calculated as the difference between the carbon and nitrogen stocks of the original, natural system (i.e. natural forest or natural grassland) and those of the cropland. GHGs from three different pools were considered: biomass carbon, soil organic carbon (SOC), and soil nitrogen. Spatially‐explicit biomass carbon stocks of natural forests at a ~1 km by ~1 km resolution were collected from Gibbs, Yui, and Plevin (2014), and default biomass carbon stocks of different types of natural grasslands were collected from Ruesch and Gibbs (2008). The biomass carbon stock of the crops was set at zero, which is similar to previous work (Elshout et al., 2015). Spatially‐explicit SOC stocks for both natural forests and croplands at a ~1 km by ~1 km resolution were also collected from Gibbs et al. (2014). The SOC stocks for natural grasslands were calculated for 18 agro‐ecological zones (AEZs) around the globe as a function of soil carbon concentration, bulk density, and depth (as per Guo & Gifford, 2002) using data from the Harmonized World Soil Database (Fischer et al., 2008). The GLC2000 land‐cover map (Bartholome & Belward, 2005) was used to identify natural grassland areas. Finally, the average natural grassland SOC stock was calculated for each of the AEZs. The change in soil nitrogen was directly related to the change in soil carbon and was calculated using the equation from Flynn et al., (2012). All SOC values were based on the top 30 cm of soil.

### 2.5 Other GHG emissions

CO_2_, N_2_O and CH_4_ emissions during the biofuel production processes were collected from the ecoinvent database (Weidema et al., 2013; Wernet et al., 2016). This included emissions from both production and application of various inputs, such as pesticides, irrigation water, and machinery use during farming and refining. Country‐specific data were preferred, but for missing countries global or *rest‐of‐the‐world* data were used. Direct and indirect emissions from nitrogen fertilizer application were calculated separately using the methods from Shcherbak, Millar, and Robertson (2014) and the IPCC (2006), respectively. The amount of nitrogen fertilizer applied was collected from Elshout et al. (2015). In order to convert quantities of N_2_O and CH_4_ to CO_2_‐equivalents, they were multiplied by their respective global warming potentials (GWPs) of 265 and 30 kg CO_2_eq kg^−1^, respectively, in the case of the 100‐year time horizon (IPCC, 2013) and 79 and 5 kg CO_2_eq kg^−1^, respectively, in the case of the 1,000‐year time horizon (Huijbregts et al., 2016). The impacts of biogenic GHGs and nonbiogenic GHGs were all considered equal (as per Hanssen, Duden, Junginger, Dale, & Hilst, 2017). However, the biogenic GHGs emitted upon combustion of the biofuel are not considered, given that the atmospheric residence time of these GHG can be considered net zero when the biofuel is produced from annual crops (Cherubini, Peters, Berntsen, Strømman, & Hertwich, 2011). An overview of data collected from the ecoinvent database can be found in Table S2.

### 2.6 Biodiversity impacts related to habitat loss

The BFs for both land occupation and land transformation were collected from Chaudhary and Brooks (2018), who calculated at an ecoregion level the average global impact of transforming and occupying annual croplands on species of all terrestrial taxa (mammals, birds, amphibians, reptiles and vascular plants) relative to the total species richness of these taxa across the globe. Their factors are calculated by combining a Species‐Area‐Relationship model with the affinity to broad land use types of 22 386 species of mammals, birds and amphibians from the IUCN Red List Habitat Classification Scheme (IUCN, 2015) and reptile and plant data from Newbold et al. (2015). The use of such Species‐Area‐Relationship‐based BFs to calculate the biodiversity impact of land use associated with a products’ life cycle was recently recommended by the UNEP‐SETAC life cycle initiative (Teixeira et al., 2016; UNEP, 2017). We determined which ecoregion each grid cell with feedstock cultivation was located in and selected the corresponding BFs (see Table S3). Chaudhary and Brooks (2018) distinguish between three farming intensity‐levels, and we used data for minimal use for the low input—no irrigation scenario, and data for intense use for both high input scenarios.

### 2.7 Biodiversity impacts related to water stress

For all feedstocks grown under high input—irrigated management, the biodiversity impact of water stress was accounted for. As spatially‐explicit data on water use by croplands was lacking, we used water consumption data from ecoinvent (Weidema et al., 2013). Only the water used during feedstock cultivation was considered, given that water withdrawn during feedstock‐to‐biofuel processing is minimal compared to water usage for irrigation (Mielke, Diaz Anadon, & Narayanamurti, 2010). Country‐specific impact factors for water stress were collected from LC‐IMPACT ( http://www.lc-impact.eu; Verones et al., 2016) (Table S4). These factors account for the relative species loss of freshwater species, terrestrial species living in river sheds, and terrestrial vascular plant species outside the wetlands.

### 2.8 Biodiversity impacts related to GHG emissions

The IAGTP was set at 4.76 10^−14^°C yr kg CO_2_eq^−1^ for a 100‐year time horizon, based on Joos et al. (2013). For the effect factor, we used data from Urban (2015), who predicts that temperatures 0.8°C above preindustrial levels will cause the extinction of 2.8% of the terrestrial species and that temperatures 4.3°C above preindustrial levels cause the extinction of 15.7% of the terrestrial species. An effect factor of 0.037 PDF °C^−1^ Celsius was calculated from the differences between these two scenarios, i.e., an average of 3.7% global species loss is expected per degree Celsius global mean temperature rise. Combining the IAGTP from Joos et al. (2013) and the effect factor from Urban (2015), we derived a BF_GHG_ of 1.76 10^−15^ PDF yr kg CO_2_eq^−1^. Using the same approach and data sources, a BF_GHG_ of 1.57 10^−14^ PDF yr kg CO_2_eq^−1^ was calculated for the 1,000‐year time horizon.

### 2.9 Reference calculations

The biodiversity impact of producing and combusting fossil fuels (*I_f,w_*) was calculated as a reference to the impact of producing biofuels. GHG emissions (from combustion as well as e.g. mining and refining of the crude oil), habitat loss (due to land transformation and occupation) and water stress (mostly due to cooling water extraction) were included in the calculations:

$$I_{f,w}=\left(M_{GHG,w}∙{BF}_{GHG}\right)+\left(A_{trans,w}∙{BF}_{HL,trans}\right)+\left(A_{occ,w}∙{BF}_{HL,occ}\right)+\left(W_{w}∙{BF}_{WS}\right)$$

where the type of fossil fuel *w* was petrol or diesel, as a reference to bioethanol and biodiesel, respectively; *M*_GHG_ is the GHG emission during fossil fuel production and combustion (in kg CO_2_eq MJ^−1^); *A_trans_* is the area of land transformation required for fossil fuel production (in m^2^ MJ^−1^); *A*_occ_ is the land area occupied for fossil fuel production (in m^2^·yr MJ^−1^); and *W* is the amount of water used during fossil fuel production (in m^3^ MJ^−1^). Area‐weighted global averages of the biodiversity impact factors of habitat loss were provided by Chaudhary (personal communication; 30–04–2018), and those for water use were collected from LC‐IMPACT ( http://www.lc-impact.eu; Verones et al., 2016). Data on GHG emissions, land use and water use for the production and combustion of petrol and diesel were collected from the ecoinvent database (Weidema et al., 2013; Wernet et al., 2016) and its documentation (Jungbluth, 2007). The GWPs mentioned above were used to convert emissions of N_2_O and CH_4_ to CO_2_‐equivalents. No by‐products of fossil fuel production were considered.

### 2.10 Fuel blends

Default calculations were performed for the production of pure bioethanol and biodiesel. However, biofuels are most often used in blends with petrol and diesel at varying mixing ratios, such as E25 (25 vol% bioethanol, 75 vol% petrol) commonly used in Brazil (Macedo, Seabra, & Silva, 2008), and B5 (5 vol% biodiesel, 95 vol% diesel) in the EU (Kousoulidou, Fontaras, Ntziachristos, & Samaras, 2010). We therefore calculated the global relative species loss related to the production of the most common fuel blends (*I_x+w_*), i.e., E10, E25, E85, B5 and B20, as follows:

$$I_{x+w,i,j}=\frac{\varphi_{BF}\times {EC}_{x}\times \rho_{x}\times \left(I_{occ,x,i,j}+I_{trans,x,i,j}\right)+\varphi_{\mathrm{FF}}\times {EC}_{w}\times \rho_{w}\times I_{fossil,w}}{\varphi_{BF}\times {EC}_{x}\times \rho_{x}+\varphi_{\mathrm{FF}}\times {EC}_{w}\times \rho_{w}}$$

where *φ* is the volume fraction of biofuel and fossil fuel in the fuel blend, and *ρ* is the fuel density (in L kg^−1^). Data on fossil fuel and biofuel densities were collected from Atabani et al. (2012) and Yüksel and Yüksel (2004), and can be found in Table S5. Impacts on the global biodiversity were calculated per liter of fuel, rather than per MJ, in order to avoid uncertainty from mixed fuel energy contents. Potential impacts of the blending process were not covered in the calculations.

### 2.11 Variable importance

We determined to what extent the variation in biodiversity impact was attributable to the producing country, crop type, farm management strategy, plantation time, and time horizon of choice by using an ANOVA on the log‐transformed biodiversity impact values. The unexplained variance (i.e., residual) can be attributed to the remaining spatial variation in biodiversity impacts within countries.

## 3 RESULTS

### 3.1 1.1. Biofuels versus fossil fuels

**Figure 1:**
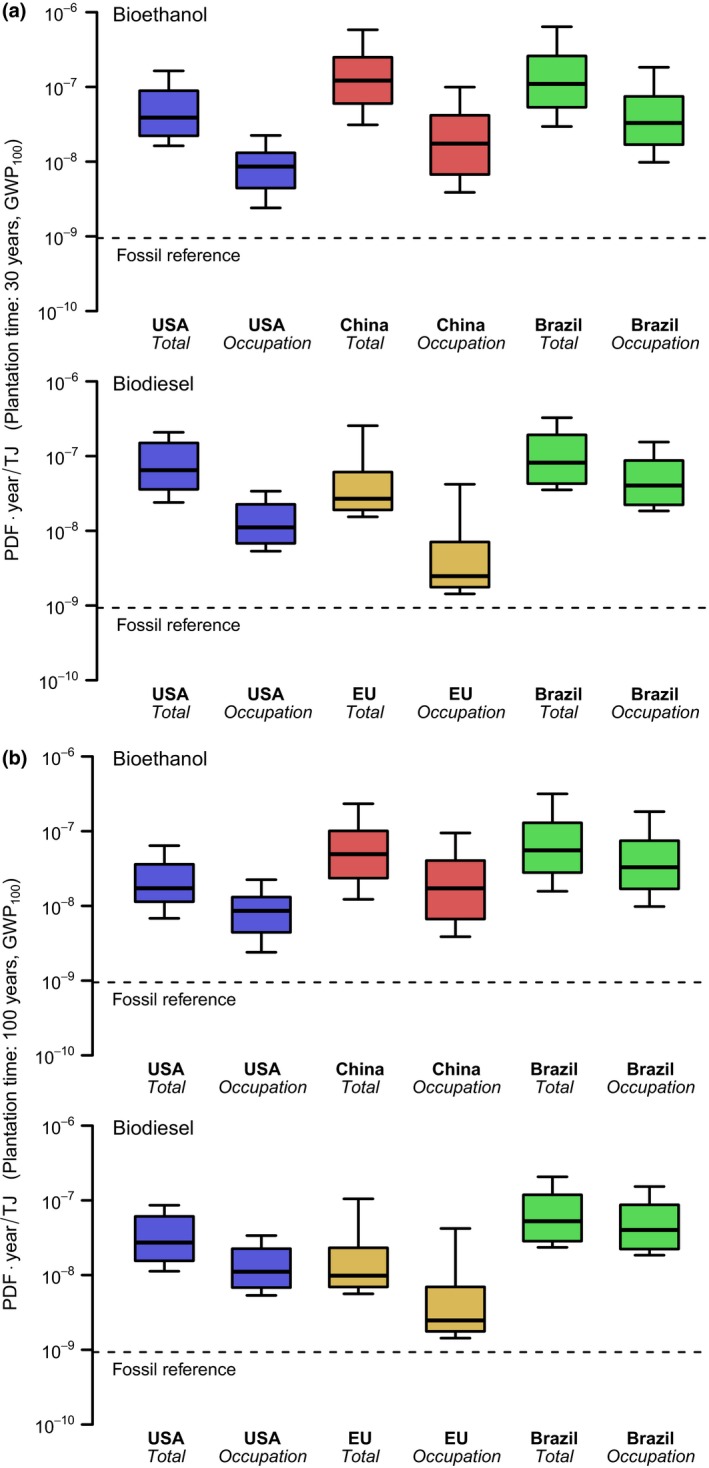
Global relative species loss due to bioethanol and biodiesel production when adopting a plantation time of (a) 30 years and (b) 100 years, and considering GHG impacts over a 100‐year time horizon. The total impact is the sum of the impacts of occupation (also provided separately) and transformation in that country. The boxes show the first quartile, median, and third quartile, and the ends of the whiskers show the 10th and 90th percentiles of the grid‐specific impacts. The dashed line shows the impact of the fossil alternatives, i.e., petrol (upper graph) and diesel (lower graph)

**Figure 2:**
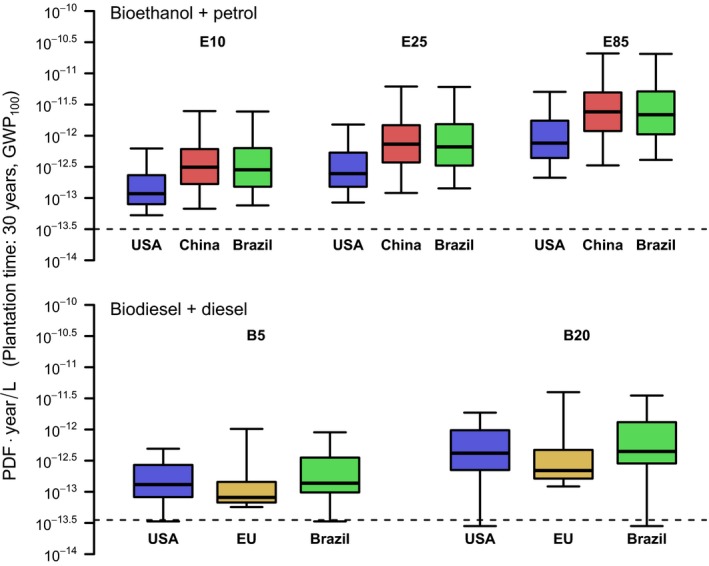
Global relative species loss due to production of various common fossil fuel‐biofuel blends, when adopting a plantation time of 30 years and considering GHG impacts over a 100‐year time horizon. Only combined impacts of occupation and transformation are shown. The boxes show the first quartile, median, and third quartile, and the ends of the whiskers show the 10th and 90th percentiles of the grid‐specific impacts. Results for other scenarios can be found in Figure S3a‐c

The occupation and transformation impact of biofuel production on global relative species loss was calculated for a total of 35,699 grid cells in the main biofuel‐producing countries. Overall, the global relative species loss caused by bioethanol and biodiesel production systems turned out to be larger than the global relative species loss caused by fossil diesel and petrol production in more than 90% of the locations. Replacing fossil fuels with biofuels would on average increase the time‐integrated global relative species loss by a factor of 30–128. Neglecting land transformation and only accounting for land occupation (referring to situations where feedstocks are grown on already established croplands), biodiversity impact of biofuel production still exceeds the impact of fossil fuel production (Figure 1). Bioethanol produced from Chinese corn and Brazilian sugarcane was found to have the largest median impact on biodiversity. The impacts of bioethanol production in these countries also showed highest spatial variation, with outcomes ranging +/‐ a factor of 19 in the case of Chinese corn and 22 in the case of Brazilian sugarcane (based on 90% range; Figure 1a). The biodiversity impacts of fuel blends increase with the share of biofuel in the mix (Figure 2). On average, B5 from European rapeseed has the smallest impact followed by E10 and B5 from USA corn and soybean, respectively. E85 from Chinese corn and Brazilian sugarcane are the worst performing fuel blends.

### 3.2 Environmental stressor importance

The impact of habitat loss due to land transformation and occupation dominates the total impact of biofuel production, as it was found to be two to three orders of magnitude higher than the impacts of water stress and GHG emissions. The biodiversity impact of water stress is found to be negligible, except for the production of corn‐based bioethanol in the USA, where it contributes more than 25% in 10% of the locations (Figure S1a). When neglecting the impact of land transformation, the biodiversity impact of land occupation is still dominant for all biofuel production systems (Figure S1b).

### 3.3 Variable importance

Country and management type were found to explain 17% and 11% of the variance, respectively, while the other variables explain less than 5% (Table S6). The residual represents the spatial variation within countries, and attributes to 67% of the variance. This indicates that the environmental performance of biofuels would improve more by selecting the most suitable locations within the countries currently producing biofuels than by switching to production in other countries, adopting different farm management strategies, growing crops for a longer time period, or approaching the impact of GHG emissions in an alternative way.

### 3.4 Sensitivity analysis

When assuming a plantation time of 100 years, the impact of land transformation is distributed over a larger amount of crop harvested, which lowers the median global relative species loss per TJ of bioenergy produced by a factor of 1.6–2.7 (Figure 1b). On the other hand using a 1,000‐year time horizon as the starting point for the time‐integrated impact of GHG emissions hardly changes the median global relative species loss of the biofuel production systems (Figure S2a) owing to the negligible contribution of GHG emissions to the total impact. However, the impacts of fossil petrol and diesel production more than doubled in case of a 1,000‐year time horizon, which caused the land occupation impacts in about 25% of the European rapeseed‐producing locations to become lower than the total impact of fossil petrol production. The same holds for the extreme scenario with a 1,000‐year time horizon for GHG impacts and a plantation time of 100 years (Figure S2b).

## 4 DISCUSSION

We show that potential global species loss per unit of first‐generation biofuel production for transport exceeds the biodiversity impacts of their fossil counterparts. The models used in the present study come, however, with a number of limitations. First, all feedstocks were assumed to be solely mono‐cropped; however, many farmers use multi‐cropping systems. For example, approximately one‐third of the farmlands in the Midwest USA alternates between corn and soybean biannually (sometimes also including other crops, such as wheat or alfalfa) (Borchers, Truex‐Powell, Wallander, & Nickerson, 2014; Plourde, Pijanowski, & Pekin, 2013). In this situation, the overall impact of bioethanol and biodiesel production would equal the average of the impacts of USA corn and USA soybean. Alternatively, multi‐cropping within 1 year would lower the impact of land transformation, as impacts are allocated among more crop biomass in the same number of years. A complete investigation of the effect of crop rotation on the relative global species loss exceeded the scope of this study, but it could potentially entail an increase in crop yield, greater soil carbon and soil nitrogen storage, and less fertilizer application, compared to a situation of mono‐cropping. Whether or not this would sufficiently improve the performance of the first‐generation biofuels to outperform fossil fuels should be investigated in future work.

Second, our outcomes rely heavily on the data input, such as the crop yields simulated by SPAM ( http://mapspam.info). Recently, Anderson, You, Wood, Wood‐Sichra, and Wu (2014) analysed four major agricultural models including SPAM, and identified considerable differences in crop yields. Still, as there is no clear preference for any alternative model, we consider SPAM as appropriate for the purpose of the current work, especially given the useful disaggregation in three farm management systems it provides.

Third, while the present study bases the biodiversity impacts of land use, water stress and climate change on recent, scientifically acclaimed and, to our opinion, most suitable methods, the biodiversity loss factors are not without uncertainty. For land use, this is demonstrated by the fact that the land use impact factors from Chaudhary and Brooks (2018) differ two orders of magnitude from those derived in previous work (Chaudhary et al., 2015) owing to methodological choices. The biodiversity loss factors are based on a comprehensive meta‐analysis from Urban (2015). Climatic tolerance of species is, however, difficult to quantify, and evolutionary changes in populations cannot be predicted (Araújo & Rahbek, 2006). Furthermore, the meta‐regression model does not account for the fact that a response to climate change by one species will have indirect impacts on the species that depend on them (i.e., biotic interactions at the community level) (Bellard, Bertelsmeier, Leadley, Thuiller, & Courchamp, 2012). Also, the LC‐IMPACT method we applied, assumes that the species losses of the three main drivers are mutually exclusive, whereas the species lost due to the three stressors may actually partly overlap. Note, however, that given the domination of land use as stressor in the total impacts of biofuel production, the influence of the assumption of simple additive effects is relative small.

Finally, it is important to emphasize that we do not take into account any potential impacts that occur abroad due to relocation of food or feed croplands after biofuel feedstock production has replaced the local food or feed production, i.e., indirect land‐use change (Searchinger et al., 2008; Verstegen et al., 2015). In our study, we always quantify species loss of land use and GHG emissions compared to the natural state, regardless of the current land use at the location. This means that biofuel production at a certain location is always evaluated compared to the natural reference. We may underestimate global species loss due to biofuel production, in situations where biofuel production results in indirect land use change in areas with higher species richness and/or higher initial carbon stocks. This would be the case, for instance, if producing corn‐based bioethanol from the US leads to indirect agricultural land transformation in the tropical rainforest of Brazil (e.g. Keeney & Hertel, 2009).

In conclusion, the current study quantified the impact of first‐generation biofuels on biodiversity due to GHG emissions, land‐use‐induced habitat loss, and water‐use‐induced habitat loss. Our findings suggest that first‐generation biofuel production in the countries evaluated here is unfavourable compared to fossil fuel use in the transportation sector, even if the biofuel feedstocks are grown on existing cropland for a period of 100 years. Habitat loss following land transformation and occupation was found to be the dominant cause of global species loss. Hence, when aiming to protect global biodiversity, the present work suggests that policy makers should support the development of other renewable energy sources with lower land demand than first‐generation biofuels, such as third‐generation biofuels (Correa, Beyer, Possingham, Thomas‐Hall, & Schenk, 2017). Further research is required to assess the biodiversity impacts of other renewable energy sources for the transport sector.
