# Supplementary material for: Global relative species loss due to first‐generation biofuel production for the transport sector
Source: Glob Change Biol Bioenergy. 2019 Mar 6;11(6):763–72. doi: 10.1111/gcbb.12597 (PMC6686982; doi:10.1111/gcbb.12597)
Supplement: Supplementary file 1 [file GCBB-11-763-s001.docx]

**Global relative species loss due to first generation biofuel production**

Pieter M.F. Elshout^1^*, Rosalie van Zelm^1^, Marijn van der Velde^2^, Zoran Steinmann^1^, Mark A. J. Huijbregts^1^

^1^Radboud University Nijmegen, Institute for Water and Wetland Research, Department of Environmental Science, PO Box 9010, 6500 GL Nijmegen, The Netherlands.

^2^European Commission, Joint Research Centre, 21027 Ispra, Italy.

*Corresponding author: [p.elshout@science.ru.nl](mailto:p.elshout@science.ru.nl)

**Supporting Information**

1. Methodology details

**Table S1**. Allocation factors for the most important by-products of the four biofuel production systems, based on the market values of the main products and by-products. We assumed that farm management did not influence the ratio between main products and by-products, so the same factors were used for all three management strategies.

| feedstock | phase^1^ | by-product(s) | use(s) | allocation factor (%)^2^ | source |
| --- | --- | --- | --- | --- | --- |
| corn | cultivation | stover | animal feed | 12 | Luo, et al. ^1^ |
| corn | processing | DDGS | animal feed | 23 | Wang, et al. ^2^ |
| rapeseed | cultivation | straw | animal feed | 3 | Bernesson ^3^ |
| rapeseed | processing | meal + glycerine | animal feed + chemical | 26 | Bernesson ^3^ |
| soybean | processing | meal + glycerine | animal feed + chemical | 57 | Wang, et al. ^2^ |
| sugarcane | processing | bagasse | electricity production | 7 | Renó, et al. ^4^, Renouf, et al. ^5^ |

^1^*Cultivation* refers to by-products that are produced on the field and gathered during harvest; *Processing* refers to by-products that are produced during crop-to-biofuel processing.

^2^Factors show the percentage of emissions that are allocated to the by-product(s).

**Table S2**. Summary of data collected or derived from the ecoinvent database^6^.

| feedstock/fuel-  life cycle phase | country^1^ | GHG emissions GWP_100_  (kg CO_2_-eq MJ^-1^) | GHG emissions GWP_1000_  (kg CO_2_-eq MJ^-1^) | land occupied^2^  (m^2^year MJ^-1^) | land transformed^2^  (m^2^ MJ^-1^) | water used^3^  (m^3^ kg crop^-1^) |
| --- | --- | --- | --- | --- | --- | --- |
| corn cultivation | RoW | 1.8E-02 | 1.3E-02 | based on spatially-explicit yields | | 1.7E-01 (CHN)  1.7E-01 (USA) |
| corn processing | RoW | 2.0E-02 | 1.8E-02 |  |  | - |
| rapeseed cultivation | RoW | 1.7E-02 | 9.7E-03 |  |  | 2.3E-01 (FRA)  8.4E-02 (GER)  1.5E-01 (RoW) |
| rapeseed processing | RoW | 8.1E-03 | 7.3E-03 |  |  | - |
| soybean cultivation | RoW | 1.3E-02 | 5.9E-03 |  |  | 1.7E-03 (BRA)  9.1E-03 (USA) |
| soybean processing | RoW | 1.1E-02 | 1.0-02 |  |  | - |
| sugarcane cultivation | Brazil | 4.6E-03 | 2.5E-03 |  |  | 1.9E-02 (BRA) |
| sugarcane processing | Brazil | 2.1E-02 | 2.0E-02 |  |  | - |
| petrol production | EU | 1.8E-02 | 1.6E-02 | 2.9E-04 | 3.1E-05 | 1.6E-04 |
| petrol combustion | EU | 7.0E-02 | 7.0E-02 | - | - | - |
| diesel production | EU | 1.2E-02 | 1.0E-02 | 2.8E-04 | 3.1E-05 | 1.5E-04 |
| diesel combustion | EU | 7.0E-02 | 7.0E-02 | - | - | - |

^1^RoW = Rest of the World, which is a weighted average of multiple producing countries.

^2^Over 30 categories of land use are distinguished in ecoinvent.

^3^Calculated as the net difference between the amount of surface water that is withdrawn and the amount of water that flows back to the surface water.

**Table S3**. Ecoregions located in the biofuel producing countries, with their respective land occupation and land transformation biodiversity impact factors (BF) from Chaudhary and Brooks ^7^. Ecoregion codes are in accordance with Olson, et al. ^8^

| country | ecoregion | code | BF_occ_  minimal use  (PDF / m^2^) | BF_occ_  intense use  (PDF / m^2^) | BF_trans_  minimal use  (PDF∙year / m^2^) | BF_trans_  intense use  (PDF∙year / m^2^) |
| --- | --- | --- | --- | --- | --- | --- |
| Austria | Central European mixed forests  Pannonian mixed forests  Western European broadleaf forests  Alps conifer and mixed forests | PA0412  PA0431  PA0445  PA0501 | 1.50E-14  1.92E-14  1.63E-14  7.90E-14 | 1.75E-14  2.28E-14  1.90E-14  9.39E-14 | 6.40E-12  8.16E-12  6.92E-12  3.03E-11 | 7.42E-12  9.68E-12  8.07E-12  3.59E-11 |
| Brazil | Araucaria moist forests  Atlantic Coast restingas  Bahia coastal forests  Bahia interior forests  Caatinga Enclaves moist forests  Caqueta moist forests  Guayanan Highlands moist forests  Guianan moist forests  Gurupa varzea  Iquitos varzea  Japura-Solimoes-Negro moist forests  Jurua-Purus moist forests  Madeira-Tapajos moist forests  Marajo Varzea forests  Maranhao Babacu forests  Mato Grosso tropical dry forests  Monte Alegre varzea  Negro-Branco moist forests  Northeastern Brazil restingas  Parana-Paraiba interior forests  Pernambuco coastal forests  Pernambuco interior forests  Purus varzea  Purus-Madeira moist forests  Rio Negro campinarana  Serra do Mar coastal forests  Solimoes-Japura moist forest  Southwest Amazon moist forests  Tapajos-Xingu moist forests  Tepuis  Tocantins-Araguaia-Maranhao moist forests  Uatuma-Trombetas moist forests  Xingu-Tocantins-Araguaia moist forests  Guianan piedmont and lowland moist forests  Atlantic dry forests  Chiquitano dry forests  Campos Rupestres montane savanna  Cerrado  Guyanan savanna  Humid Chaco  Uruguayan savanna  Pantanal  Caatinga  Alvarado mangroves  Belizean Reef mangroves | NT0101  NT0102  NT0103  NT0104  NT0106  NT0107  NT0124  NT0125  NT0126  NT0128  NT0132  NT0133  NT0135  NT0138  NT0139  NT0140  NT0141  NT0143  NT0144  NT0150  NT0151  NT0152  NT0156  NT0157  NT0158  NT0160  NT0163  NT0166  NT0168  NT0169  NT0170  NT0173  NT0180  NT0182  NT0202  NT0212  NT0703  NT0704  NT0707  NT0708  NT0710  NT0907  NT1304  NT1401  NT1406 | 4.80E-13  1.52E-12  1.44E-12  6.10E-13  1.70E-13  1.56E-13  4.63E-13  2.20E-13  3.85E-13  3.43E-13  1.55E-13  1.64E-13  1.95E-13  2.07E-13  8.48E-14  1.05E-13  2.85E-13  1.74E-13  1.21E-13  3.82E-13  2.50E-12  8.75E-13  1.89E-13  1.73E-13  1.71E-13  2.28E-12  2.00E-13  2.82E-13  1.71E-13  1.06E-13  1.60E-13  1.50E-13  1.55E-13  2.45E-13  1.91E-13  1.71E-13  9.70E-13  1.09E-13  2.14E-13  1.03E-13  1.47E-13  9,49E-14  9.99E-14  4.05E-13  2.24E-12 | 4.89E-13  1.78E-12  1.50E-12  6.21E-13  1.97E-13  1.65E-13  4.77E-13  2.25E-13  4.20E-13  3.50E-13  1.60E-13  1.68E-13  1.98E-13  2.15E-13  8.82E-14  1.07E-13  2.96E-13  1.79E-13  1.31E-13  3.88E-13  2.71E-12  8.97E-13  1.93E-13  1.77E-13  1.76E-13  2.39E-12  2.07E-13  2.87E-13  1.75E-13  1.07E-12  1.64E-13  1.54E-13  1.59E-13  2.50E-13  1.99E-13  1.75E-13  9.77E-13  1.10E-13  2.16E-13  1.04E-13  1.48E-13  9.58E-14  1.01E-13  4.12E-13  2.28E-12 | 4.07E-11  1.26E-10  1.22E-10  5.21E-11  1.44E-11  1.32E-11  3.91E-11  1.87E-11  3.23E-11  2.91E-11  1.32E-11  1.40E-11  1.68E-11  1.77E-11  7.24E-12  8.95E-12  2.42E-11  1.48E-11  1.03E-11  3.26E-11  2.08E-10  7.39E-11  1.61E-11  1.47E-11  1.46E-11  1.93E-10  1.70E-11  2.40E-11  1.46E-11  8.94E-11  1.37E-11  1.28E-11  1.32E-11  2.08E-11  1.70E-11  1.54E-11  6.11E-11  6.86E-12  1.34E-11  6.53E-12  9.29E-12  8.57E-12  6.56E-12  5.06E-11  2.80E-10 | 4.14E-11  1.47E-10  1.27E-10  5.30E-11  1.66E-11  1.40E-11  4.03E-11  1.92E-11  3.51E-11  2.97E-11  1.36E-11  1.43E-11  1.70E-11  1.83E-11  7.52E-12  9.12E-12  2.51E-11  1.52E-11  1.11E-11  3.31E-11  2.25E-10  7.57E-11  1.65E-11  1.51E-11  1.50E-11  2.02E-10  1.75E-11  2.45E-11  1.49E-11  9.08E-11  1.40E-11  1.31E-11  1.35E-11  2.12E-11  1.78E-11  1.58E-11  6.15E-11  6.92E-12  1.35E-11  6.57E-12  9.35E-12  8.65E-12  6.61E-12  5.15E-11  2.85E-10 |
| China | Jian Nan subtropical evergreen forests  Northern Indochina subtropical forests  South China-Vietnam subtropical evergreen forests  Hainan Island monsoon rain forests  South Taiwan monsoon rain forests  Taiwan subtropical evergreen forests  Eastern Himalayan broadleaf forests  Eastern Himalayan subalpine conifer forests  Gizhou Plateau broadleaf and mixed forests  Yunnan Plateau subtropical evergreen forests  Central China loess plateau mixed forests  Changbai Mountains mixed forests  Changjiang Plain evergreen forests  Daba Mountains evergreen forests  Huang He Plain mixed forests  Manchurian mixed forests  Northeast China Plain deciduous forests  Qin Ling Mountains deciduous forests  Sichuan Basin evergreen broadleaf forests  Altai montane forest and forest steppe  Da Hinggan-Dzhagdy Mountains conifer forests  Helanshan montane conifer forests  Hengduan Mountains subalpine conifer forests  Northeastern Himalayan subalpine conifer forests  Nujiang Langcang Gorge alpine conifer and mixed forests  Qilian Mountains conifer forests  Qionglai-Minshan conifer forests  Tian Shan montane conifer forests  East Siberian taiga  Altai steppe and semi-desert  Daurian forest steppe  Emin Valley steppe  Mongolian-Manchurian grassland  Tian Shan foothill arid steppe  Amur meadow steppe  Bohai Sea saline meadow  Nenjiang River grassland  Ussuri-Wusuli meadow and forest meadow  Yellow Sea saline meadow  Altai alpine meadow and tundra  Central Tibetan Plateau alpine steppe  Eastern Himalayan alpine shrub and meadows  Karakoram-West Tibetan Plateau alpine steppe  Ordos Plateau steppe  Qilian Mountains subalpine meadow  Southeast Tibet shrublands and meadow  Tian Shan montane steppe and meadow  Tibetan Plateau alpine shrublands and meadows  Western Himalayan alpine shrub and Meadows  Yarlung Zambo arid steppe  Alashan Plateau semi-desert  Eastern Gobi desert steppe  Junggar Basin semi-desert  Qaidam Basin semi-desert  Taklimakan desert | IM0118  IM0137  IM0149  IM0169  IM0171  IM0172  IM0401  IM0501  PA0101  PA0102  PA0411  PA0414  PA0415  PA0417  PA0424  PA0426  PA0430  PA0434  PA0437  PA0502  PA0505  PA0508  PA0509  PA0514  PA0516  PA0517  PA0518  PA0521  PA0601  PA0802  PA0804  PA0806  PA0813  PA0818  PA0901  PA0902  PA0903  PA0907  PA0908  PA1001  PA1002  PA1003  PA1006  PA1013  PA1015  PA1017  PA1019  PA1020  PA1021  PA1022  PA1302  PA1314  PA1317  PA1324  PA1330 | 1.33E-13  3.99E-13  2.80E-13  1.35E-12  4.54E-12  1.68E-12  7.01E-13  6.66E-13  1.71E-13  2.34E-13  5.53E-14  4.42E-14  9.42E-14  1.58E-13  4.56E-14  4.88E-14  3.65E-14  2.03E-13  1.46E-13  2.90E-14  1.77E-14  1.64E-13  3.64E-13  6.16E-13  6.74E-13  2.47E-13  8.79E-13  6.65E-14  8.87E-15  3.03E-14  2.15E-14  2.43E-14  1.90E-14  3.46E-14  2.28E-14  2.26E-14  2.59E-14  4.93E-14  3.01E-13  2.77E-14  2.03E-14  1.79E-13  7.21E-14  2.72E-14  2.59E-14  1.02E-13  3.10E-14  3.13E-14  9.09E-14  5.69E-14  1.33E-14  1.29E-14  1.95E-14  1.34E-14  1.16E-14 | 1.44E-13  4.24E-13  3.06E-13  1.48E-12  5.67E-12  1.87E-12  7.50E-13  7.15E-13  1.82E-13  2.50E-13  6.00E-14  4.81E-14  1.01E-13  1.68E-13  4.93E-14  5.20E-14  3.93E-14  2.19E-13  1.55E-13  3.18E-14  1.91E-14  1.70E-13  3.98E-13  6.51E-13  7.31E-13  2.60E-13  9.51E-13  7.76E-14  9.37E-15  3.13E-14  2.20E-14  2.51E-14  1.94E-14  3.64E-14  2.36E-14  2.42E-14  2.75E-14  5.13E-14  3.21E-13  2.87E-14  2.05E-14  1.83E-13  7.30E-14  2.79E-14  2.63E-14  1.03E-13  3.24E-14  3.17E-14  9.49E-14  5.80E-14  1.35E-14  1.31E-14  2.00E-14  1.36E-14  1.21E-14 | 1.69E-11  5.13E-11  3.59E-11  1.73E-10  5.53E-10  2.14E-10  1.83E-10  1.98E-10  4.01E-11  5.50E-11  2.32E-11  1.86E-11  3.95E-11  6.66E-11  1.92E-11  2.06E-11  1.52E-11  8.57E-11  6.10E-11  1.13E-11  6.72E-12  6.51E-11  1.38E-10  2.33E-10  2.60E-10  9.56E-11  3.34E-10  2.50E-11  5.06E-12  6.54E-12  4.59E-12  5.19E-12  4.03E-12  7.29E-12  4.50E-12  4.39E-12  5.05E-12  9.75E-12  6.18E-11  5.55E-12  4.03E-12  3.53E-11  1.46E-11  5.38E-12  5.14E-12  2.02E-11  6.13E-12  6.20E-12  1.77E-11  1.12E-11  1.95E-12  1.89E-12  2.87E-12  1.96E-12  1.69E-12 | 1.83E-11  5.45E-11  3.93E-11  1.90E-10  6.90E-10  2.38E-10  1.95E-10  2.12E-10  4.27E-11  5.87E-11  2.52E-11  2.03E-11  4.25E-11  7.06E-11  2.08E-11  2.19E-11  1.64E-11  9.22E-11  6.45E-11  1.23E-11  7.27E-12  6.74E-11  1.51E-11  2.46E-10  2.81E-10  1.00E-10  3.61E-10  2.91E-11  5.34E-12  6.75E-12  4.71E-12  5.36E-12  4.12E-12  7.64E-12  4.65E-12  4.68E-12  5.35E-12  1.01E-11  6.59E-11  5.74E-12  4.08E-12  3.59E-11  1.47E-11  5.51E-12  5.22E-12  2.04E-11  6.38E-12  6.28E-12  1.85E-11  1.14E-11  1.98E-12  1.92E-12  2.95E-12  1.98E-12  1.75E-12 |
| France | Atlantic mixed forests  Cantabrian mixed forests  Pyrenees conifer and mixed forests  Western European broadleaf forests  Alps conifer and mixed forests  Corsican montane broadleaf and mixed forests  Italian sclerophyllous and semi-deciduous forests  Northeastern Spain & Southern France Mediterranean forests  Tyrrhenian-Adriatic Sclerophyllous and mixed forests | PA0402  PA0406  PA0433  PA0445  PA0501  PA1204  PA1211  PA1215  PA1222 | 1.76E-14  7.26E-14  2.32E-13  1.63E-14  7.90E-14  1.04E-12  8.69E-14  7.53E-14  2.07E-13 | 2.07E-14  8.94E-14  2.93E-13  1.90E-14  9.39E-14  1.29E-12  9.71E-14  8.43E-14  2.44E-13 | 7.44E-12  3.07E-11  9.49E-11  6.92E-12  3.03E-11  2.04E-10  1.74E-11  1.50E-11  4.19E-11 | 8.73E-12  3.78E-11  1.20E-10  8.07E-12  3.59E-11  2.55E-10  1.94E-11  1.68E-11  4.92E-11 |
| Germany | Atlantic mixed forests  Baltic mixed forests  Central European mixed forests  Western European broadleaf forests  Alps conifer and mixed forests | PA0402  PA0405  PA0412  PA0445  PA0501 | 1.76E-14  1.10E-14  1.50E-14  1.63E-14  7.90E-14 | 2.07E-14  2.07E-14  1.75E-14  1.90E-14  9.39E-14 | 7.44E-12  4.57E-12  6.40E-12  6.92E-12  3.03E-11 | 8.73E-12  5.54E-12  7.42E-12  8.07E-12  3.59E-11 |
| Italy | Appenine deciduous montane forests  Dinaric Mountains mixed forests  Po Basin mixed forests  Alps conifer and mixed forests  Illyrian deciduous forests  Italian sclerophyllous and semi-deciduous forests  Northeastern Spain & Southern France Mediterranean forests  South Appenine mixed montane forests  Tyrrhenian-Adriatic Sclerophyllous and mixed forests | PA0401  PA0418  PA0432  PA0501  PA1210  PA1211  PA1215  PA1218  PA1222 | 8.16E-14  7.24E-14  6.10E-14  7.90E-14  1.66E-13  8.69E-14  7.53E-14  2.43E-13  2.07E-13 | 9.70E-14  8.66E-14  7.10E-14  9.39E-14  1.88E-13  9.71E-14  8.43E-14  2.82E-13  2.44E-13 | 3.42E-11  3.07E-11  2.60E-11  3.03E-11  3.31E-11  1.74E-11  1.50E-11  4.76E-11  4.19E-11 | 4.05E-11  3.66E-11  3.03E-11  3.59E-11  3.74E-11  1.94E-11  1.68E-11  5.52E-11  4.92E-11 |
| Poland | Baltic mixed forests  Central European mixed forests  Western European broadleaf forests  Carpathian montane conifer forests | PA0405  PA0412  PA0445  PA0504 | 1.10E-14  1.50E-14  1.63E-15  3.33E-14 | 2.07E-14  1.75E-14  1.90E-14  4.13E-14 | 4.57E-12  6.40E-12  6.92E-12  1.26E-11 | 5.54E-12  7.42E-12  8.07E-12  1.55E-11 |
| United States of America | Sierra Madre Occidental pine-oak forests  Sierra Madre Oriental pine-oak forests  Allegheny Highlands forests  Appalachian mixed mesophytic forests  Appalachian/Blue Ridge forests  Central U.S. hardwood forests  East Central Texas forests  Eastern forest/boreal transition  Eastern Great Lakes lowland forests  Mississippi lowland forests  New England/Acadian forests  Northeastern coastal forests  Ozark Mountain forests  Southeastern mixed forests  Southern Great Lakes forests  Upper Midwest Forest/Savanna transition zone  Western Great Lakes forests  Willamette Valley forests  Arizona Mountains forests  Atlantic coastal pine barrens  Blue Mountains forests  British Columbia mainland coastal forests  Cascade Mountains leeward forests  Central and Southern Cascades forests  Central Pacific coastal forests  Colorado Rockies forests  Eastern Cascades forests  Florida sand pine scrub  Great Basin montane forests  Klamath-Siskiyou forests  Middle Atlantic coastal forests  North Central Rockies forests  Northern California coastal forests  Northern Pacific coastal forests  Okanogan dry forests  Piney Woods forests  Puget lowland forests  Sierra Nevada forests  South Central Rockies forests  Southeastern conifer forests  Wasatch and Uinta montane forests  Alaska Peninsula montane taiga  Cook Inlet taiga  Copper Plateau taiga  Interior Alaska/Yukon lowland taiga  Western Gulf coastal grasslands  California Central Valley grasslands  Canadian Aspen forests and parklands  Central and Southern mixed grasslands  Central forest/grasslands transition zone  Central tall grasslands  Edwards Plateau savanna  Flint Hills tall grasslands  Montana Valley and Foothill grasslands  Nebraska Sand Hills mixed grasslands  Northern mixed grasslands  Northern short grasslands  Northern tall grasslands  Palouse grasslands  Texas blackland prairies  Western short grasslands  Alaska/St. Elias Range tundra  Aleutian Islands tundra  Beringia lowland tundra  Beringia upland tundra  Pacific Coastal Mountain icefields and tundra  California coastal sage and chaparral  California interior chaparral and woodlands  California montane chaparral and woodlands  Chihuahuan desert  Colorado Plateau shrublands  Great Basin shrub steppe  Mojave desert  Snake/Columbia shrub steppe  Sonoran desert  Tamaulipan mezquital  Wyoming Basin shrub steppe  South Florida rocklands  Everglades  Hawaii tropical moist forests  Hawaii tropical dry forests  Hawaii tropical high shrublands  Hawaii tropical low shrublands  **Global average**^1^ | NA0302  NA0303  NA0401  NA0402  NA0403  NA0404  NA0405  NA0406  NA0407  NA0409  NA0410  NA0411  NA0412  NA0413  NA0414  NA0415  NA0416  NA0417  NA0503  NA0504  NA0505  NA0506  NA0507  NA0508  NA0510  NA0511  NA0512  NA0513  NA0515  NA0516  NA0517  NA0518  NA0519  NA0520  NA0522  NA0523  NA0524  NA0527  NA0528  NA0529  NA0530  NA0601  NA0603  NA0604  NA0607  NA0701  NA0801  NA0802  NA0803  NA0804  NA0805  NA0806  NA0807  NA0808  NA0809  NA0810  NA0811  NA0812  NA0813  NA0814  NA0815  NA1101  NA1102  NA1106  NA1107  NA1117  NA1201  NA1202  NA1203  NA1303  NA1304  NA1305  NA1308  NA1309  NA1310  NA1312  NA1313  NT0164  NT0904  OC0106  OC0202  OC0701  OC0702  - | 3.23E-13  6.73E-13  3.84E-14  6.71E-14  1.45E-13  5.00E-14  8.64E-14  1.97E-14  3.09E-14  7.77E-14  3.00E-14  6.59E-14  1.27E-13  7.12E-14  3.84E-14  3.10E-14  2.68E-14  2.58E-13  1.35E-13  1.02E-13  6.50E-14  3.93E-14  4.43E-14  1.52E-13  1.59E-13  6.52E-14  8.49E-14  2.96E-13  2.35E-13  2.04E-13  1.13E-13  3.22E-14  2.71E-13  3.87E-14  3.80E-14  6.69E-14  1.49E-13  2.41E-13  4.04E-14  1.42E-13  6.66E-14  2.62E-14  1.69E-14  1.49E-14  1.35E-14  1.18E-13  2.68E-13  1.54E-14  5.02E-14  4.29E-14  2.79E-14  1.66E-13  4.68E-14  3.32E-14  3.77E-14  2.63E-14  2.99E-14  2.44E-14  4.33E-14  1.59E-13  4.39E-14  1.49E-14  4.08E-13  3.88E-14  5.41E-14  3.09E-14  2.71E-13  2.30E-13  3.78E-13  8.38E-14  4.68E-14  3.68E-14  7.76E-14  4.01E-14  8.52E-14  8.47E-14  3.10E-14  1.77E-12  2.73E-13  1.15E-11  5.47E-12  6.00E-12  4.09E-12  1.60E-13 | 3.50E-13  7.46E-13  4.17E-14  7.06E-14  1.49E-13  5.38E-14  9.33E-14  2.08E-14  3.32E-14  8.34E-14  3.15E-14  6.97E-14  1.33E-13  7.59E-14  4.16E-14  3.34E-14  2.83E-14  2.74E-13  1.43E-13  1.10E-13  6.94E-14  4.13E-14  4.80E-14  1.58E-13  1.64E-13  6.94E-14  9.02E-14  3.38E-13  2.55E-13  2.13E-13  1.21E-13  3.40E-14  2.85E-13  4.03E-14  4.16E-14  7.49E-14  1.58E-13  2.51E-13  4.32E-14  1.54E-13  7.18E-14  2.72E-14  1.77E-14  1.57E-14  1.38E-14  1.21E-13  2.84E-13  1.56E-14  5.18E-14  4.44E-14  2.88E-14  1.70E-13  4.95E-14  3.42E-14  3.90E-14  2.68E-14  3.04E-14  2.50E-14  4.52E-14  1.61E-13  4.52E-14  1.53E-14  4.19E-13  4.01E-14  5.59E-14  3.17E-14  2.88E-13  2.42E-13  4.07E-13  8.82E-14  4.78E-14  3.76E-14  8.10E-14  4.10E-14  8.99E-14  8.84E-14  3.17E-14  2.25E-12  3.07E-13  1.18E-11  5.76E-12  6.27E-12  4.87E-12  1.99E-11 | 6.40E-11  1.34E-10  1.65E-11  2.89E-11  6.21E-11  2.15E-11  3.72E-11  8.48E-12  1.33E-11  3.32E-11  1.29E-11  2.85E-11  5.44E-11  3.05E-11  1.65E-11  1.33E-11  1.15E-11  1.14E-10  5.07E-11  3.72E-11  2.43E-11  1.45E-11  1.63E-11  5.69E-11  5.97E-11  2.40E-11  3.16E-11  1.08E-10  8.80E-11  7.59E-11  4.16E-11  1.19E-11  1.01E-10  1.43E-11  1.39E-11  2.45E-11  5.51E-11  8.94E-11  1.50E-11  5.23E-11  2.47E-11  1.24E-11  7.97E-12  7.06E-12  6.44E-12  1.04E-11  6.06E-11  3.46E-12  1.14E-11  9.69E-12  6.29E-12  3.74E-11  1.05E-11  7.47E-12  8.48E-12  5.92E-12  6.78E-12  5.47E-12  9.76E-12  3.56E-11  9.95E-12  5.68E-12  1.58E-10  1.47E-11  2.05E-11  1.19E-11  5.48E-11  4.63E-11  7.53E-11  1.50E-11  8.41E-12  6.62E-12  1.40E-11  7.27E-12  1.53E-11  1.51E-11  5.58E-12  1.45E-10  2.39E-11  1.32E-09  4.25E-10  7.12E-10  4.73E-10  1.70E-13 | 6.93E-11  1.48E-10  1.79E-11  3.04E-11  6.37E-11  2.31E-11  4.01E-11  8.96E-12  1.42E-11  3.57E-11  1.36E-11  3.01E-11  5.67E-11  3.25E-11  1.78E-11  1.43E-11  1.21E-11  1.21E-10  5.36E-11  4.00E-11  2.59E-11  1.52E-11  1.76E-11  5.90E-11  6.18E-11  2.55E-11  3.35E-11  1.23E-10  9.54E-11  7.95E-11  4.45E-11  1.26E-11  1.06E-10  1.49E-11  1.52E-11  2.75E-11  5.84E-11  9.29E-11  1.60E-11  5.64E-11  2.66E-11  1.29E-11  8.37E-12  7.45E-12  6.60E-12  1.07E-11  6.40E-11  3.52E-12  1.17E-11  1.00E-11  6.48E-12  3.82E-11  1.11E-11  7.70E-12  8.77E-12  6.03E-12  6.89E-12  5.62E-12  1.02E-11  3.62E-11  1.02E-11  5.86E-12  1.62E-10  1.52E-11  2.12E-11  1.22E-11  5.80E-11  4.86E-11  8.08E-11  1.58E-11  8.59E-12  6.76E-12  1.46E-11  7.43E-12  1.61E-11  1.57E-11  5.70E-12  1.83E-10  2.68E-11  1.36E-09  4.47E-10  7.42E-10  5.62E-10  2.13E-11 |

^1^ global averages were provided by dr. Chaudhary (personal communication; 30-04-2018)

**Table S4.** Country-specific biodiversity impact factors for water stress (BF_WS_) from <http://lc-impact.eu> ^9^.

| country | BF_WS_  (PDF∙year / m^3^) |
| --- | --- |
| Austria | 1.60E-14 |
| Brazil | 2.76E-15 |
| China | 2.32E-15 |
| France | 6.19E-16 |
| Germany | 4.21E-15 |
| Italy | 3.41E-15 |
| Poland | 4.30E-16 |
| United States of America | 1.15E-12 |
| **Global average** | 1.63E-13 |

**Table S5.** Biofuel and fossil fuel densities, used to convert kilograms to litre before calculating the impact of fuel blends. Values are based on Atabani, et al. ^10^ and Yüksel and Yüksel ^11^.

| fuel type | kg fuel / litre |
| --- | --- |
| bioethanol | 0.79 |
| biodiesel | 0.88 |
| petrol | 0.74 |
| diesel | 0.83 |

1. Additional results

a.

*

*

b.

*

*

**Figure S1.** Contributions of water use, GHG emissions and land use to the total biodiversity impact. Contributions are shown for (a) a scenario with both land transformation and land occupation, and (b) a scenario with only occupation. The boxes show the first quartile, median, and third quartile, and the ends of the whiskers show the 10th and 90th percentiles of the grid-specific impacts.


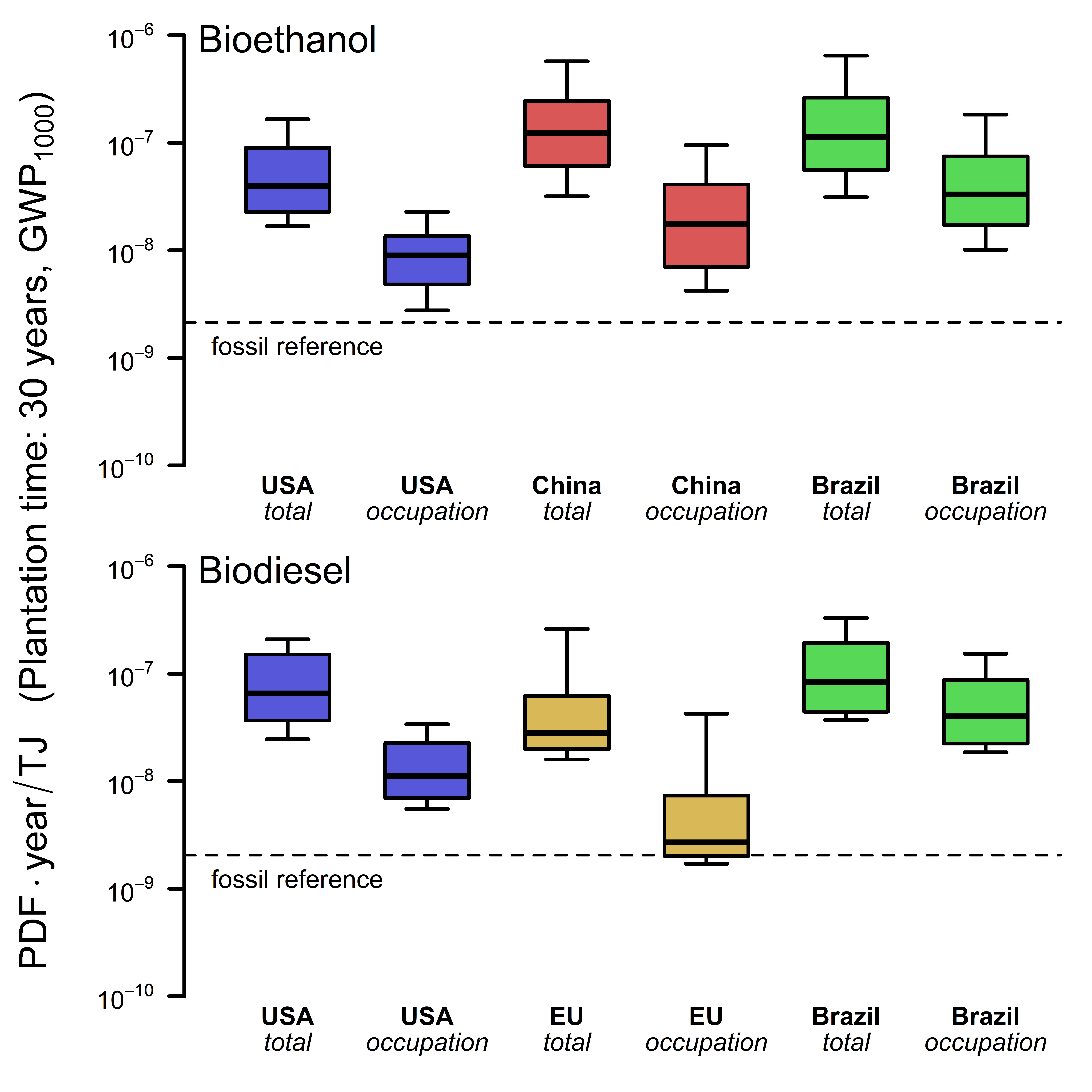
a.

b.


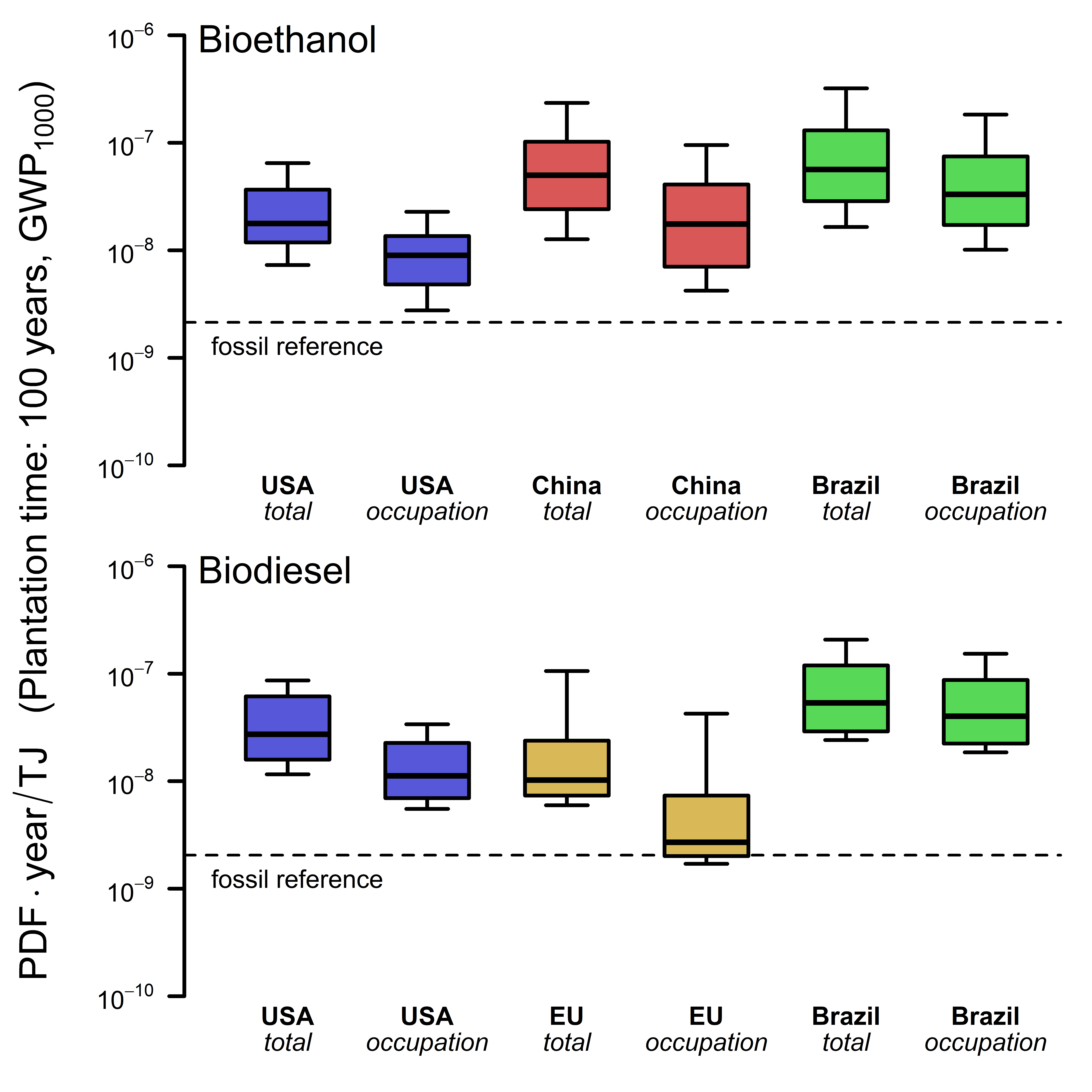


**Figure S2.** Global relative species loss due to bioethanol and biodiesel calculated for two scenarios: (a) 30-year plantation time and 1000-year time horizon; and (b) 100-year plantation time and 1000-year time horizon. Impacts are given for a scenario with both land transformation and land occupation (*total*) and a scenario with only occupation. The boxes show the first quartile, median, and third quartile, and the ends of the whiskers show the 10th and 90th percentiles of the grid-specific impacts. The dashed line shows the impact of the fossil alternatives, i.e., gasoline (upper graph) and diesel (lower graph).

a.


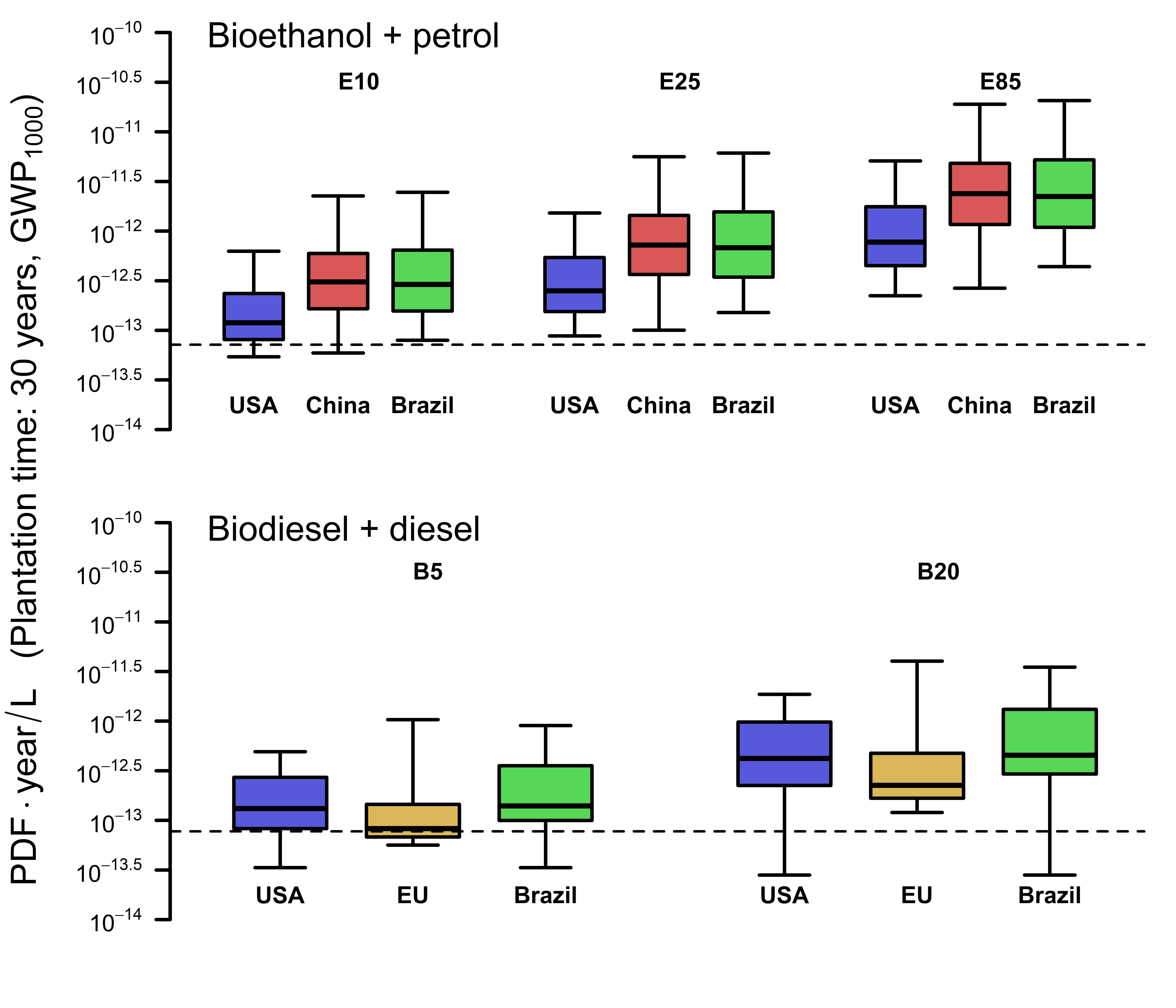


b.


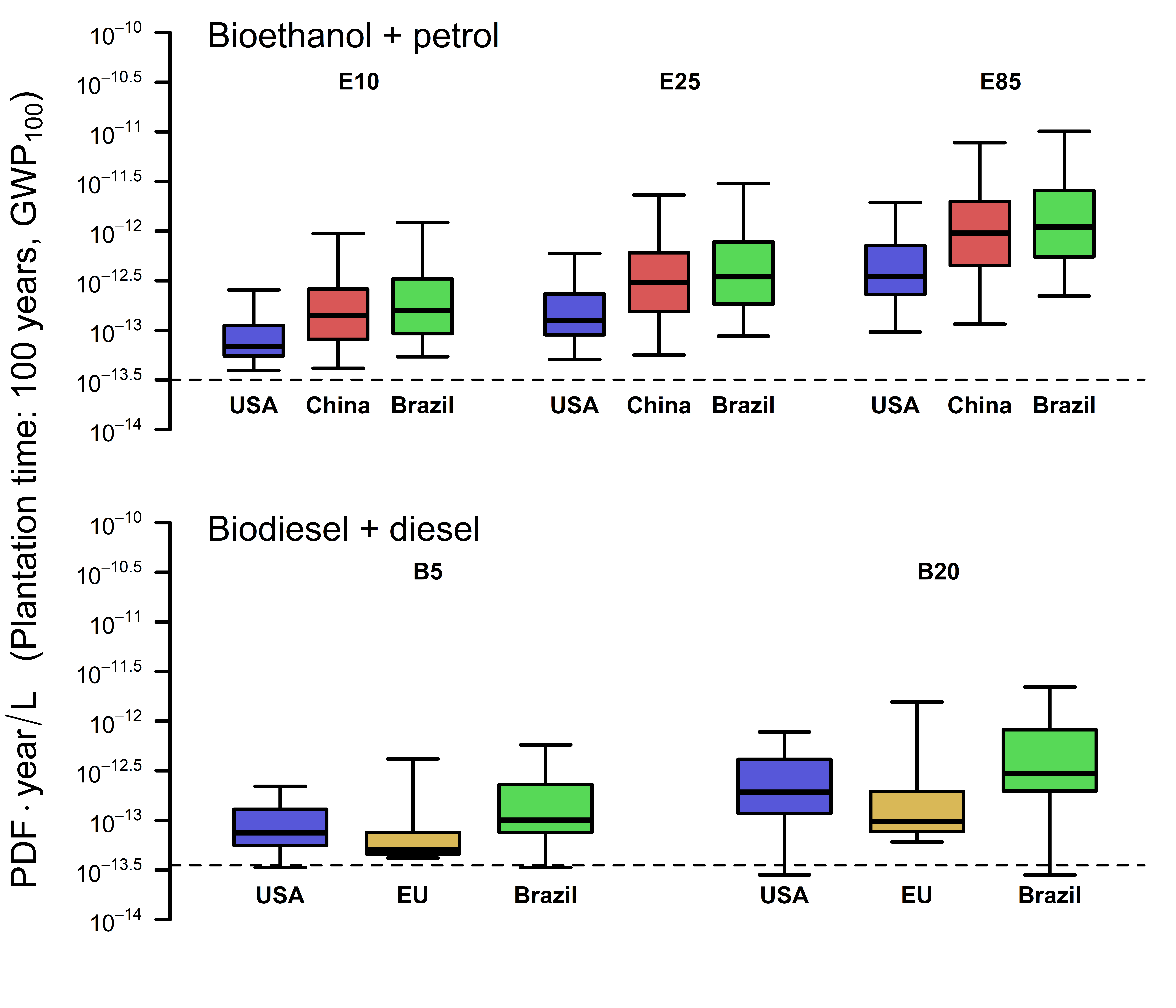


c.

**
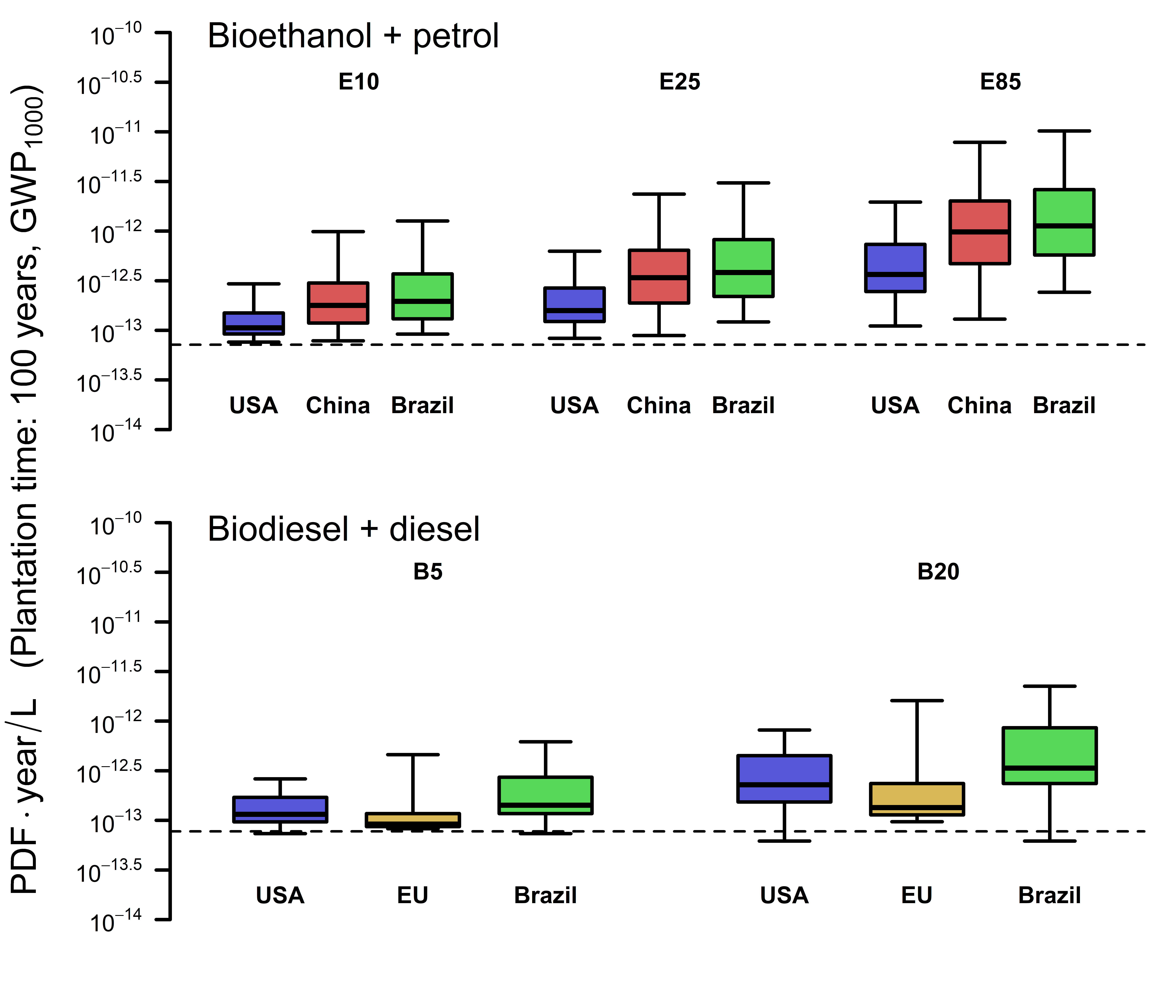
**

**Figure S3.** Global relative species loss due to production of various common fossil fuel-biofuel blends, calculated for three scenarios: (a) 30-year plantation time and 1000-year time horizon; (b) 100-year plantation time and 100-year time horizon; and (c) 100-year plantation time and 1000-year time horizon. Only combined impacts of occupation and transformation are shown. The boxes show the first quartile, median, and third quartile, and the ends of the whiskers show the 10th and 90th percentiles of the grid-specific impacts.

| variable | explained variance |
| --- | --- |
| country | 17.0% |
| crop type | 0.5% |
| management type | 10.8% |
| plantation time | 4.2% |
| time horizon | 0.3% |
| location (residual) | 67.3% |

**Table S6.** Explained variance of five variables and the residual to the total biodiversity impact, based on an ANOVA’s sum of squares.

SI References

1 Luo, L., Van der Voet, E., Huppes, G. & Udo de Haes, H. A. Allocation issues in LCA methodology: A case study of corn stover-based fuel ethanol. *International Journal of Life Cycle Assessment* **14**, 529-539 (2009).

2 Wang, M., Huo, H. & Arora, S. Methods of dealing with co-products of biofuels in life-cycle analysis and consequent results within the U.S. context. *Energy Policy* **39**, 5726-5736 (2011).

3 Bernesson, S. Life cycle assessment of rapeseed oil, rape methyl ester and ethanol as fuels: A comparison between large- and small-scale production. (Sveriges lantbruksuniversiteit, Uppsala, 2004).

4 Renó, M. L. G. *et al.* A LCA (life cycle assessment) of the methanol production from sugarcane bagasse. *Energy* **36**, 3716-3726, doi:<https://doi.org/10.1016/j.energy.2010.12.010> (2011).

5 Renouf, M. A., Pagan, R. J. & Wegener, M. K. Life cycle assessment of Australian sugarcane products with a focus on cane processing. *The International Journal of Life Cycle Assessment* **16**, 125-137, doi:10.1007/s11367-010-0233-y (2011).

6 Weidema, B. P. *et al.* Overview and methodology. Data quality guideline for the ecoinvent database version 3. (The Ecoinvent Centre, St. Gallen, 2013).

7 Chaudhary, A. & Brooks, T. M. Land Use Intensity-Specific Global Characterization Factors to Assess Product Biodiversity Footprints. *Environmental Science & Technology*, doi:10.1021/acs.est.7b05570 (2018).

8 Olson, D. M. *et al.* Terrestrial ecoregions of the world: A new map of life on Earth. *Bioscience* **51**, 933-938 (2001).

9 Verones, F. *et al.* LC-IMPACT version 0.5: A spatially differentiated life cycle impact assessment approach. (2016).

10 Atabani, A. E. *et al.* A comprehensive review on biodiesel as an alternative energy resource and its characteristics. *Renewable and Sustainable Energy Reviews* **16**, 2070-2093, doi:<https://doi.org/10.1016/j.rser.2012.01.003> (2012).

11 Yüksel, F. & Yüksel, B. The use of ethanol–gasoline blend as a fuel in an SI engine. *Renewable Energy* **29**, 1181-1191, doi:<https://doi.org/10.1016/j.renene.2003.11.012> (2004).
